# Construction and validation of a machine learning-based nomogram model for predicting pneumonia risk in patients with catatonia: a retrospective observational study

**DOI:** 10.3389/fpsyt.2025.1557659

**Published:** 2025-03-14

**Authors:** Yi-chao Wang, Qian He, Yue-jing Wu, Li Zhang, Sha Wu, Xiao-jia Fang, Shao-shen Jia, Fu-gang Luo

**Affiliations:** Affiliated Mental Health Center & Hangzhou Seventh People's Hospital, Zhejiang University School of Medicine, Hangzhou, Zhejiang, China

**Keywords:** catatonia, pneumonia, clinical prediction model, machine learning, nomogram

## Abstract

**Objective:**

Catatonia was often complicated by pneumonia, and the development of severe pneumonia after admission posed significant challenges to its treatment. This study aimed to develop a Nomogram Model based on pre-admission characteristics of patients with catatonia to predict the risk of pneumonia after admission.

**Methods:**

This retrospective observational study reviewed catatonia patients hospitalized at Hangzhou Seventh People’s Hospital from September 2019 to November 2024. Data included demographic characteristics, medical history, maintenance medications, and pre-admission clinical presentations. Patients were divided into catatonia with and without pneumonia groups. The LASSO Algorithm was used for feature selection, and seven machine learning models: Decision Tree(DT), Logistic Regression(LR), Naive Bayes(NB), Random Forest(RF), K Nearest Neighbors(KNN), Gradient Boosting Machine(GBM), Support Vector Machine(SVM) were trained. Model performance was evaluated using AUC, Accuracy, Sensitivity, Specificity, Positive Predictive Value, Negative Predictive Value, F1 Score, Cohen’s Kappa, and Brier Score, and Brier score. The best-performing model was selected for multivariable analysis to determine the variables included in the final Nomogram Model. The Nomogram Model was further validated through ROC Curves, Calibration Curves, Decision Curve Analysis (DCA), and Bootstrapping to ensure discrimination, calibration, and clinical applicability.

**Results:**

Among 156 patients, 79 had no pneumonia, and 77 had pneumonia. LASSO Algorithm identified 15 non-zero coefficient variables (LASSO 1-SEλ=0.076). The GBM showed the best performance (AUC = 0.954, 95% CI: 0.924-0.983, *vs* other models by DeLong’s test: *P* < 0.05). Five key variables: Age, Clozapine, Diaphoresis, Intake Refusal, and Waxy Flexibility were used to construct the Nomogram Model. Validation showed good discrimination (AUC = 0.803, 95% CI: 0.735-0.870), calibration, and clinical applicability. Internal validation (Bootstrapping, n=500) confirmed model stability (AUC = 0.814, 95% CI: 0.743-0.878; Hosmer-Lemeshow *P* = 0.525).

**Conclusion:**

This study developed a Nomogram Model based on five key factors, demonstrating significant clinical value in predicting the risk of pneumonia in hospitalized patients with catatonia.

## Introduction

Catatonia was a group of severe psychomotor syndromes characterized by impaired motor, language, and complex behavior. It usually presented with rigidity, decreased activity, mutism, defecation, and eating disorders. In recent years, the DSM-5 included catatonia as a separate diagnostic item, rather than just as part of the schizophrenia spectrum (1). This change was also followed in the ICD-11, with a significant change in the definition of catatonia compared to the ICD-10, reclassifying it as a separate syndrome rather than a specific subtype of mental disorder (2). Catatonia was a relatively common syndrome. In a study of 130 patients in an acute psychiatric lockdown unit, 16.9% were diagnosed with catatonia (3). In a meta-analysis of 74 catatonia studies, the mean prevalence of catatonia was 9.0%. The highest prevalence of catatonia was found in non-psychiatric outpatients or inpatients (15.8%), while the lowest prevalence was found in psychiatric outpatients (3.2%). The prevalence of catatonia in the presence of a somatic or neurological condition but no comorbid psychiatric condition was 20.6%, compared with 5.7% in the sample with mixed psychiatric conditions (4). However, the relatively high incidence of catatonia was accompanied by a high risk. Patients with catatonia were often accompanied by a variety of complications, including pneumonia, dehydration, urinary tract infection, urinary retention, pressure ulcers, rhabdomyolysis, renal failure, sepsis, deep vein thrombosis, and pulmonary embolism, etc (5, 6).

Among them, pneumonia was one of the most troublesome complications of catatonia. Patients with catatonia were prone to develop aspiration pneumonia and acute respiratory failure due to increased catatonic muscle tone, weakened swallowing and cough reflexes, prolonged bed rest, and the use of sedative drugs. These complications obviously hindered the treatment of catatonia, leading to poor prognosis and even life-threatening conditions (7). In 2018, Japanese scholars compared 1719 patients with schizophrenia and found that the in-hospital mortality of patients in the catatonic group (140 cases) (7.1%) was significantly higher than that in the non-catatonic group (1579 cases) (1.6%). Among them, 4 patients in the catatonic group died of pneumonia and respiratory failure (8). During the treatment of catatonia, the presence of pneumonia made the treatment more complicated. On the one hand, if catatonia combined with pneumonia was not treated in time, pneumonia could further worsen, leading to severe pneumonia, respiratory failure, systemic infection, and even sepsis and septic shock. On the other hand, the use of benzodiazepines and electroconvulsive therapy (ECT) could cause excessive sedation and respiratory depression, further aggravating pneumonia. Studies showed that the use of ECT and restraint protection in specialized psychiatric hospitals significantly increased the risk of lower respiratory tract infections (LRI) and pneumonia, especially in patients with schizophrenia (9). The use of these treatments in patients with catatonia presented clinicians with a dilemma when treating catatonia and managing complications of pneumonia (10, 11).

Clinically, patients with catatonia were prone to aspiration due to poor posture and impaired swallowing function caused by behavioral limitations, and the long-term use of sedatives might have inhibited respiratory function (8). These factors might have made patients more susceptible to pneumonia. Especially after patients were transferred from outpatient to inpatient settings, their mobility was further reduced, and the risk of pneumonia might have significantly increased. However, to date, there was a lack of effective methods to systematically assess these risks, particularly a lack of systematic quantitative prediction tools. This left clinicians facing challenges in early risk assessment and individualized management, unable to effectively predict which patients were more likely to develop pneumonia, thus missing the opportunity for preventive intervention. Currently, only one retrospective study explored the risk factors for pneumonia in patients with catatonia (12). However, this study did not further develop a predictive model, which limited its clinical applicability and practical utility. Existing studies primarily focused on the risk factors for pneumonia in other mental disorders, such as schizophrenia, depression, and bipolar disorder (13–15). However, the clinical characteristics, medication use, and risk of complications in patients with these mental disorders were significantly different from those of patients with catatonia, making the existing prediction tools difficult to meet the needs of this special population. Therefore, the development of specific predictive tools for patients with catatonia was essential to reduce the incidence of pneumonia and improve clinical outcomes.

Based on this clinical need, this study aimed to construct a set of nomogram prediction models through retrospective analysis to assess the risk of pneumonia in outpatients with catatonia after hospitalization. The establishment of a prediction model for the risk of pneumonia in patients with catatonia after hospitalization not only helped to improve the prognosis of patients but also provided a scientific basis for medical staff to make clinical decisions. The nomogram, as an intuitive and easy-to-use tool, provided a systematic and quantitative method for medical staff to quickly determine the risk of pneumonia according to the individual situation of patients and to take corresponding early preventive measures in time. In addition, this study provided new insights for further research on the complications of catatonia and promoted the optimization of systematic management strategies for catatonia. A retrospective observational design was used in this study, and the patients with catatonia were divided into two groups: with and without pneumonia. The differences in various influencing factors between the two groups were analyzed, and then a nomogram model was constructed. Statistical methods were used to screen and model these factors to ensure the scientific rigor and accuracy of the prediction model.

## Methods

### Research subjects and sample size estimation

**Figure 1:**
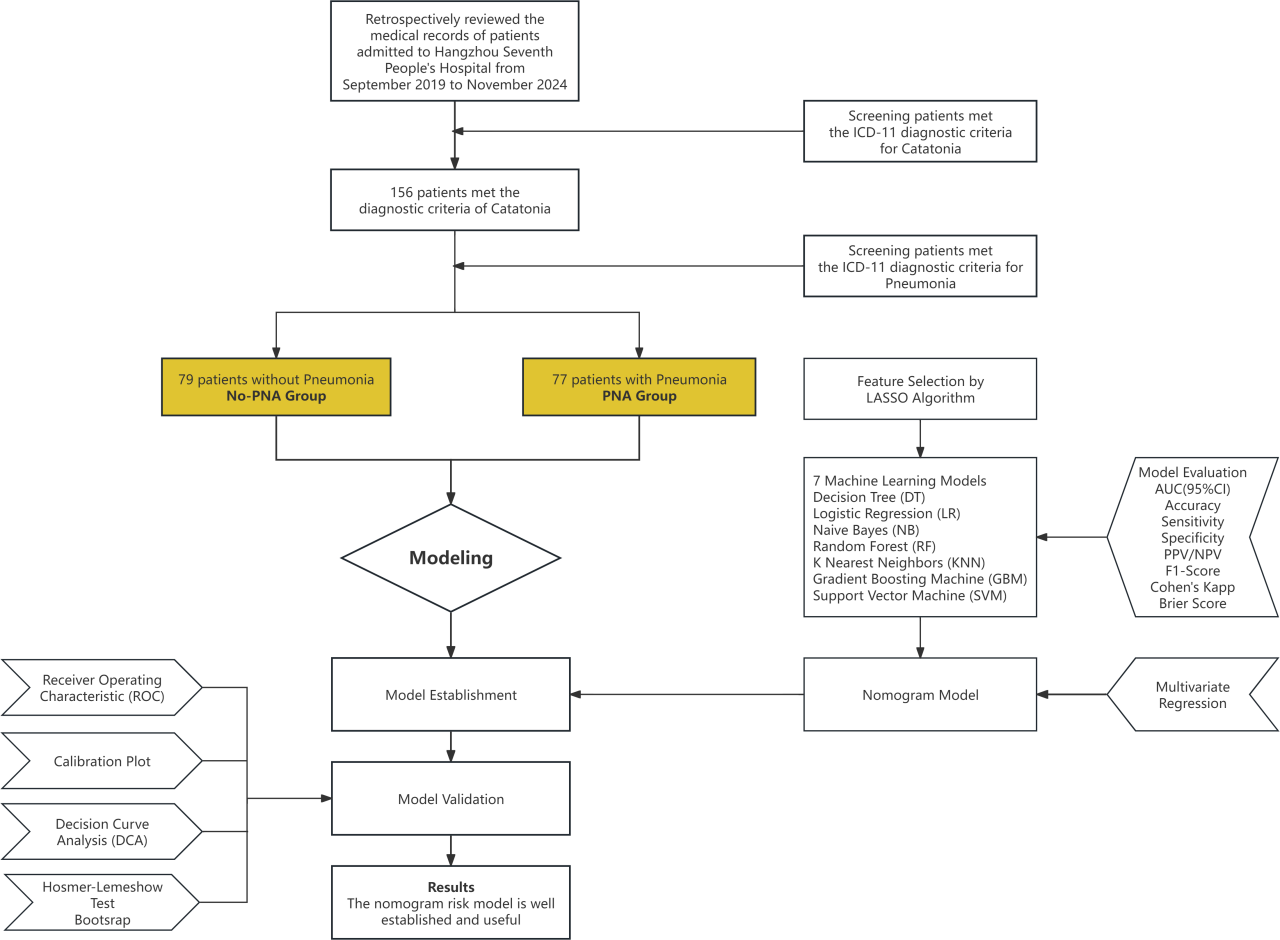
Flow chart.

This study was a retrospective observational study. Clinical data were collected through the electronic medical record system from September 2019 to November 2024 at Hangzhou Seventh People’s Hospital (a psychiatric specialty hospital). The data included pre-admission general clinical information of patients with catatonia who were hospitalized in the outpatient and emergency departments, general psychiatry, clinical psychology, geriatric psychiatry, and intensive care units. The collected information covered demographic characteristics, medical history, maintenance medications, and pre-admission clinical presentations. Patients were divided into two groups based on the presence of pneumonia: catatonia without pneumonia(No-PNA Group) and catatonia with pneumonia(PNA Group). The LASSO algorithm was used for feature selection, and machine learning models were applied to further screen key features. A nomogram prediction model was subsequently constructed (**Figure 1**). This study was a retrospective observational study that reviewed general clinical data without any experimental interventions. Ethical approval was obtained from the Ethics Committee of Hangzhou Seventh People’s Hospital.

This study used the “pmsampsize” package in R to estimate the required sample size to ensure the stability and reliability of the prediction model. Considering that cases of catatonia were relatively rare and often accompanied by pneumonia, the number of variables included in the prediction model was set to 5. The probability of positive events was set to 0.4, and the c-statistic was set to 0.8 (AUC = 0.8). The calculation yielded a Cox-Snell R-squared value of 0.2521, with an allowable difference of 0.05 between the apparent R-squared and the adjusted R-squared, and an allowable error of 0.05 for the intercept estimate. Given the rarity of catatonia cases, the study adopted LASSO regularization, which allowed the selection of the minimum sample size and the lowest EPP value. However, it still adhered to the 10 EPV principle, meaning that the number of positive events per predictor variable needed to be ≥10. The R output was as follows: Samp_size: Minimum required sample size was 153, Shrinkage: 0.900, Maximum R-squared: 0.2521, Nagelkerke R-squared: 0.341, EPP: The number of positive events per predictor variable was 12.24. Based on these sample size estimates, a total of 160 subjects were planned to be included to ensure the robustness and scientific validity of the model.

### ICD-11 criteria for catatonia

Inpatient and outpatient medical records were screened using the ICD-11 diagnostic criteria for catatonia. The presence of three or more of the following symptoms of decreased, increased, or abnormal psychomotor activity was required. The three symptoms could have come from one or any combination of the following three symptom clusters: Decreased Psychomotor Activity: Staring, ambitendency, negativism, stupor, and mutism. Increased Psychomotor Activity: Extreme hyperactivity or agitation for no reason with nonpurposeful movements and/or uncontrollable, extreme emotional reactions, impulsivity, and combativeness. Abnormal Psychomotor Activity: Grimacing, mannerisms, posturing, stereotypy, rigidity, echophenomena, verbigeration, waxy flexibility, and catalepsy (2).

### ICD-11 criteria for pneumonia

The diagnosis of pneumonia primarily relied on clinical symptoms, physical signs, and auxiliary examinations, which included: Clinical Symptoms: Common symptoms included fever, cough (which might have been accompanied by sputum or blood-streaked sputum), chest pain, and shortness of breath. Physical Signs: Abnormal findings on lung auscultation included crackles and/or diminished breath sounds. Imaging Studies: Chest X-rays or computed tomography (CT) scans were crucial for diagnosing pneumonia. These imaging techniques typically revealed infiltrative shadows in the lungs, indicative of pneumonia. Microbiological Tests: These included sputum culture, blood culture, and throat swabs. Blood Tests: There might have been an increase in white blood cell count or abnormal white blood cell differential, as well as elevated C-reactive protein (CRP), which were indicators of inflammation (2).

### Observation characteristics

Demographic Characteristics: This included gender, age, height, weight, BMI, marital status, educational level, and social adaptability (e.g., unemployment and school dropout status). Social Support: Information about parents and children, living arrangements (e.g., independent living, residence in long-term care facilities, or bedridden status), rural residency, medical insurance coverage, and details about referrers and guardians were collected. Substance Dependence: Past and current smoking and alcohol consumption were assessed. History of Physical Illnesses: This included previous diagnoses related to cardiovascular, respiratory, digestive, renal, metabolic, and neurological systems. History of Psychiatric Disorders and Catatonia: Information was gathered about family history of psychiatric disorders, age at onset, duration of illness, number of previous hospitalizations, previous episodes of catatonia, hospitalizations due to catatonia, and hospitalizations for catatonia complicated by pneumonia. Maintenance Medication Status: Details about the use of antipsychotic medications, benzodiazepines, and other drugs were recorded, with specific dose conversions included. The dosage of antipsychotic drugs: Antipsychotic medications used by the patients were converted into equivalent olanzapine doses (16). The dosage of benzodiazepines: Benzodiazepines used by patients were converted into equivalent doses of diazepam (17). Current Catatonia Episode: This included the time of consultation, multiple transfers, cross-regional medical treatment, presence of stress events, drug discontinuation, or overdose. Basic vital signs (such as heart rate, blood pressure, and respiratory rate) and physical symptoms (such as feeding status, constipation, sweating, pressure ulcers, and weight changes) were also recorded. Diagnosis and Symptoms of Catatonia: The specific manifestations and diagnostic criteria of catatonia in the patients were evaluated. It is important to note that the above observational indicators did not include parameters explicitly indicating infection, such as body temperature, white blood cell count, C-reactive protein (CRP), or chest CT findings.

### Catatonia assessment with BFCSI

This study adopted the Bush Francis Catatonia Screening Instrument (BFCSI) to assess catatonia symptoms. The Bush Francis Catatonia Rating Scale (BFCRS) was a 23-item rating scale that collected data through observation, physical examination, and interviews. It was used for the systematic, standardized, and quantitative assessment of catatonia. Among the 23 items in the BFCRS, Bush et al. identified the first 14 as the most common and typical symptoms of catatonia. These items formed the BFCSI, which was used to screen for the presence of catatonia. If two or more items in the BFCSI were rated as “present,” catatonia was considered, regardless of severity. The screening section marked items 1-14 as either “present” or “absent” (18, 19). We chose BFCSI instead of BFCRS as the assessment scale for catatonia, primarily based on the needs of our study design. Since this study aimed to screen variables based on pre-admission clinical characteristics to predict the risk of pneumonia in hospitalized patients with catatonia, rapid screening was more important and easier to implement than detailed scoring in pre-admission settings such as outpatient and emergency departments. Therefore, we ultimately selected BFCSI as the assessment tool.

### Statistical analysis and LASSO for feature selection

This study utilized R software (version 4.4.2; R Foundation for Statistical Computing, Vienna, Austria; available at https://www.r-project.org) for statistical analysis. Continuous variables were first tested for normality. Those that followed a normal distribution were expressed as mean ± standard deviation (x ± s) and were compared between groups using the independent sample T-test. Non-normally distributed continuous variables were expressed as median (interquartile range) and were compared using the Mann-Whitney U test. Categorical variables were presented as counts (percentages) and were analyzed with the Pearson χ² test. Statistical significance was set at *P* < 0.05. The least absolute shrinkage and selection operator (LASSO) regression analysis was used for dimensionality reduction and selection of important features. The “glmnet” package was applied to fit the data using the glm function, with the family argument set to binomial, and the summary function was used to examine the coefficients and their p-values. The optimal tuning parameter λ was determined through 10-fold cross-validation, and the non-zero variables corresponding to 1-SE λ were finally selected for inclusion in the model.

### Machine learning model construction and evaluation

Seven Machine Learning algorithms were applied to establish models. The algorithms and their implementations in R were as follows: Decision Tree (DT): Models were constructed using the “rpart” package with the rpart() function. Parameters were tuned using the “caret” package. Logistic Regression (LR): Models were implemented with the glm() function in the “stats” package. The family argument was set to binomial for binary classification. Naive Bayes (NB): Models were built using the naiveBayes() function in the “e1071” package. The Laplace smoothing parameter was tuned to improve performance. Random Forest (RF): Models were developed with the randomForest() function in the “randomForest” package. The number of trees (ntree) and variables randomly sampled at each split (mtry) were optimized through grid search. K Nearest Neighbors (KNN): Models were implemented using the knn() function in the “class” package. The number of neighbors (k) was optimized using cross-validation. Gradient Boosting Machine (GBM): Models were constructed with the gbm() function in the “gbm” package. Hyperparameters, including learning rate, number of trees, and interaction depth, were tuned via grid search. Support Vector Machine (SVM): Models were developed using the svm() function in the “e1071” package. Kernel functions (linear, radial basis function) and cost parameters were optimized through tune.svm() for performance improvement.

All models were trained and optimized using 10-fold cross-validation and were evaluated based on the following performance metrics. The “pROC” package was employed to plot Receiver Operating Characteristic (ROC) curves and calculate the Area Under the Curve (AUC) to assess the discriminative ability of the models. Additionally, the models were further evaluated based on Accuracy, Sensitivity, Specificity, Positive Predictive Value (PPV), Negative Predictive Value (NPV), F1-Score, Cohen’s Kappa, and Brier Score. The Delong Test was used to compare and validate the performance of the models, ultimately identifying the optimal model. To further explain the model predictions and the contribution of each variable to the model output, SHAP (SHapley Additive exPlanations) value analysis was employed. SHAP values were calculated using the “iml” package in R, and visualizations were generated with the “shapviz” and “fastshap” packages. Three types of SHAP plots were used to intuitively interpret the model results: Beeswarm Plot: The SHAP summary plot was used to illustrate the global impact of each variable on the model output. This plot displayed the distribution of SHAP values for each variable, highlighting both the magnitude and direction of their effects, which helped analyze the overall contribution of variables in the model. Feature Importance Bar Plot: Variables were ranked based on the mean absolute SHAP values, generating a feature importance plot. This visualization emphasized the overall impact of each variable and aided in identifying key predictors driving the model’s performance. Waterfall Plot: The SHAP waterfall plot was used to explain individual predictions by showing how each variable contributed positively or negatively to the final prediction for a specific sample. It highlighted the additive nature of SHAP values, helping to understand the contribution and interactions of variables in single-sample predictions. This analytical approach not only revealed the global feature importance within the model but also provided a detailed explanation of individual prediction patterns, ensuring both interpretability and clinical utility of the model results.

### Nomogram model construction and validation

Based on the variables selected from the optimal machine learning model, multivariable regression analysis was conducted to further refine key variables and construct a nomogram model. The final nomogram model underwent comprehensive evaluation, including plotting the ROC curve and calculating the AUC to assess discriminative ability (20), generating a calibration curve to evaluate calibration, and performing the Hosmer-Lemeshow test to verify the model’s goodness-of-fit (21). Additionally, decision curve analysis (DCA) was used to assess the clinical utility of the model (22). To ensure the robustness and reliability of the model, bootstrap resampling (n = 500) was applied for internal validation. Finally, the included variables were subjected to Variance Inflation Factor (VIF) testing to detect potential multicollinearity issues. The construction and validation of the model follow and are evaluated based on the Transparent Reporting of a Multivariable Prediction Model for Individual Prognosis or Diagnosis (TRIPOD) guidelines (23). A dynamic nomogram was developed using the “DynNom” and “Shiny” packages in R and deployed as a web application for online access.

## Results

**Figure 2:**
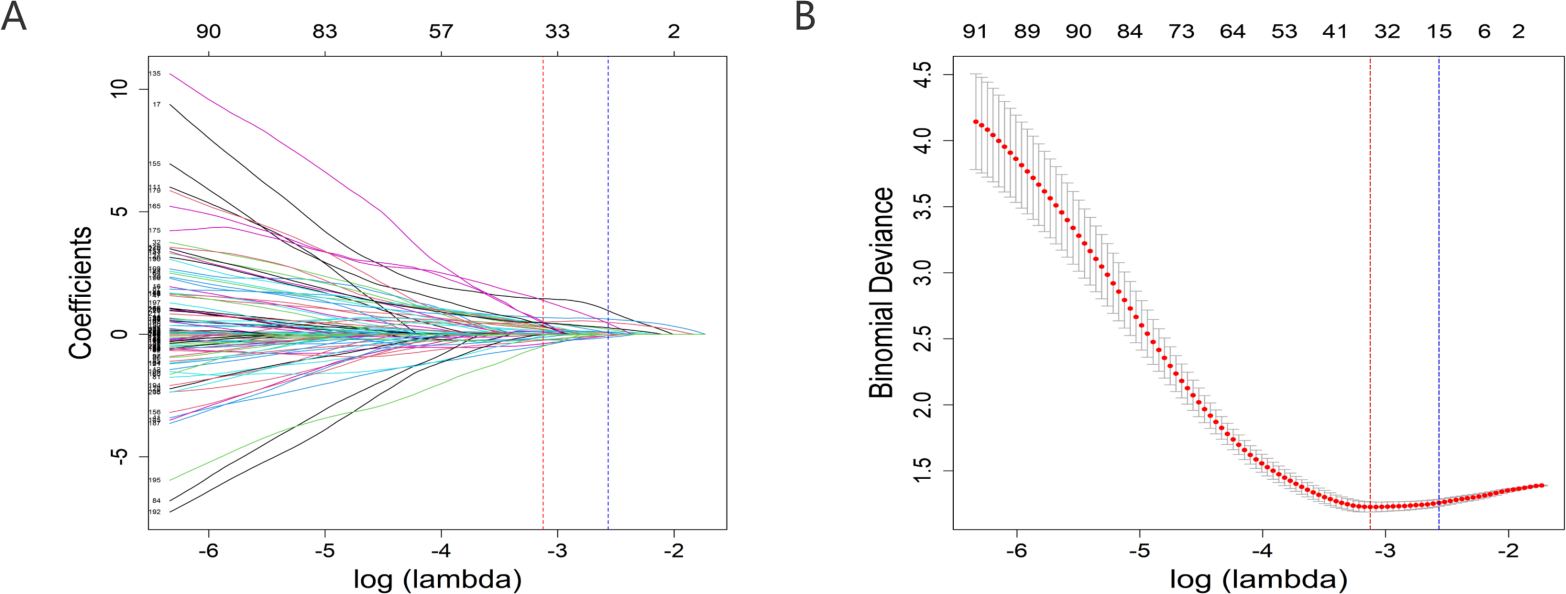
Predictor selection using the LASSO regression analysis with 10-fold cross-validation. **(A)** A coefficient profile plot was created against the log (lambda) sequence. **(B)** Tuning parameter (lambda) selection of deviance in the LASSO regression based on the minimum criteria (left dotted line) and the 1-SE criteria (right dotted line). lambda.min=0.04392175; lambda.1se=0.07675451. In the present study, predictor's selection was according to the 1-SE criteria (right dotted line), where 15 nonzero coefficients were selected. LASSO, least absolute shrinkage and selection operator; SE, standard error.

A total of 156 patients met the ICD-11 diagnostic criteria for catatonia. According to the ICD-11 criteria for pneumonia, there were 79 patients in the catatonia without pneumonia group and 77 patients in the catatonia with pneumonia group. The patient dataset included 213 variables, with most continuous variables categorized based on clinically relevant cutoff values commonly used in practice. In this study, there were no missing values in the data. Univariate analyses were conducted using T-tests, U-tests, and chi-square tests, identifying 46 variables with *P*-values less than 0.05 (**Supplementary Materials**). LASSO regression was employed for variable selection, and 15 non-zero coefficient variables were identified at λ 1SE (λ = 0.07675451) (**Figures 2A, B**). These variables included Age, BMI, Widowed, Dropout, Mental Disorder History (1-5 years), Previous Hospitalizations due to Catatonia with Pneumonia, Clozapine, MECT History, Intake Refusal, Constipation, Diaphoresis, No Somatic Disease History, Arrhythmia History, Digestive System History, and BFCSI Waxy Flexibility.

**Figure 3:**
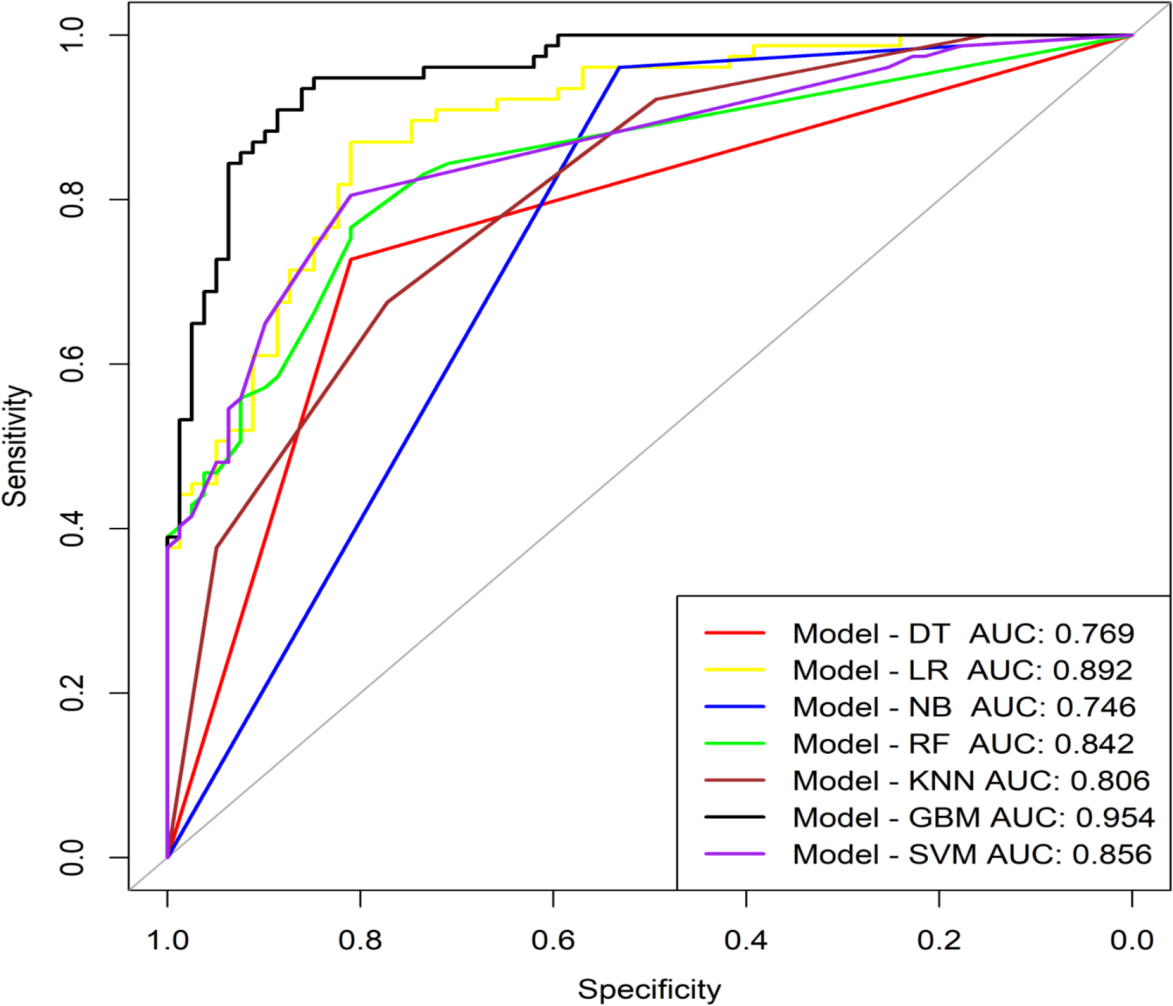
ROC curve analysis of machine learning algorithms for prediction of the risk of Catatonia-Pneumonia. DT, Decision Tree; LR, Logistic Regression; NB, Naive Bayes; RF, Random Forest; KNN, K Nearest Neighbors;GBM, Gradient Boosting Machine; SVM, Support Vector Machine; AUC, Area Under Curve.

Subsequently, seven Machine Learning methods were used to construct predictive models. The GBM model performed the best, achieving an AUC (95% CI) of 0.954 (0.924-0.983) (**Figure 3**), an Accuracy of 0.885, a Sensitivity of 0.896, a Specificity of 0.873, a Positive Predictive Value (PPV) of 0.873, a Negative Predictive Value (NPV) of 0.896, an F1-Score of 0.885, Cohen’s Kappa of 0.769, and a Brier Score of 0.100. Furthermore, DeLong’s test was used to compare the ROC curves of the GBM model with those of other models, and the results all showed *P* < 0.05, indicating that the GBM model significantly outperformed all other models (**Tables 1**). In R, the gbm() function was used to train the model, and variable importance was assessed using the summary function. Relative Influence (rel.inf) values were calculated to measure each variable’s relative contribution to the predictive outcome. Eleven variables with rel.inf > 1 were selected, including: BMI, Age, Clozapine, Diaphoresis, BFCSI Waxy Flexibility, Digestive System History, Constipation, MECT History, Intake Refusal, Mental Disorder History (1-5 years), and No Somatic Disease History (**Tables 2**).

**Table 1:** Diagnostic performance of seven machine learning classifiers.

Model	AUC (95% CI)	Z	*P* Value	Accuracy	Sensitivity	Specificity	PPV	NPV	F1-Score	Cohen’s Kapp	Brier Score
DT	0.769 (0.702-0.835)	6.175	<0.001*	0.769	0.727	0.810	0.789	0.753	0.757	0.538	0.174
LR	0.892 (0.842-0.941)	2.924	0.003*	0.801	0.753	0.848	0.829	0.779	1.000	0.602	0.139
NB	0.746 (0.687-0.806)	7.371	<0.001*	0.744	0.961	0.532	0.667	0.933	0.770	0.452	0.208
RF	0.842 (0.782-0.901)	3.286	0.001*	0.782	0.753	0.810	0.795	0.771	0.773	0.923	0.056
KNN	0.806 (0.741-0.870)	4.071	<0.001*	0.724	0.772	0.675	0.709	0.743	0.739	0.448	1.247
GBM	0.954 (0.924-0.983)	–	–	0.885	0.896	0.873	0.873	0.896	0.885	0.769	0.100
SVM	0.856 (0.799-0.913)	2.963	0.003*	0.808	0.805	0.810	0.805	0.810	0.805	0.615	1.148

DT, Decision Tree; LR, Logistic Regression; NB, Naive Bayes;RF, Random Forest; KNN, K Nearest Neighbors; GBM, Gradient Boosting Machine; SVM, Support Vector Machine.

*DeLong’s test was used to compare the ROC curves of the GBM model with those of other models, and the results all showed P < 0.05, indicating statistically significant differences.

**Table 2:** Multivariate logistic regression analysis.

Variable	rel.inf Value	VIF Value	Beta	OR	95%CI	*P*-value
(Intercept)	–	–	-4.769	0.008	0.00 -0.111	0.001
BMI	30.731	1.026	0.074	1.077	0.989 -1.178	0.093
Age	28.548	1.107	0.024	1.024	1.003 -1.047	0.028*
Diaphoresis	7.717	1.051	1.265	3.542	1.389 -9.702	0.010*
Clozapine	7.219	1.180	1.046	2.845	1.082 -7.936	0.038*
BFCSI Waxy Flexibility	5.085	1.130	1.147	3.148	1.079 -10.101	0.042*
Digestive System History	4.522	1.083	–	–	–	–
Constipation	4.192	1.168	–	–	–	–
MECT History	3.444	1.141	0.750	2.117	0.762 -6.183	0.156
Intake Refusal	3.301	1.103	1.335	3.801	1.286 -12.691	0.021*
Mental Disorder History (1-5 years)	2.653	1.150	–	–	–	–
No Somatic Disease History	2.102	1.115	-1.058	0.347	0.093 -1.133	0.092

*P<0.05.

**Figure 4:**
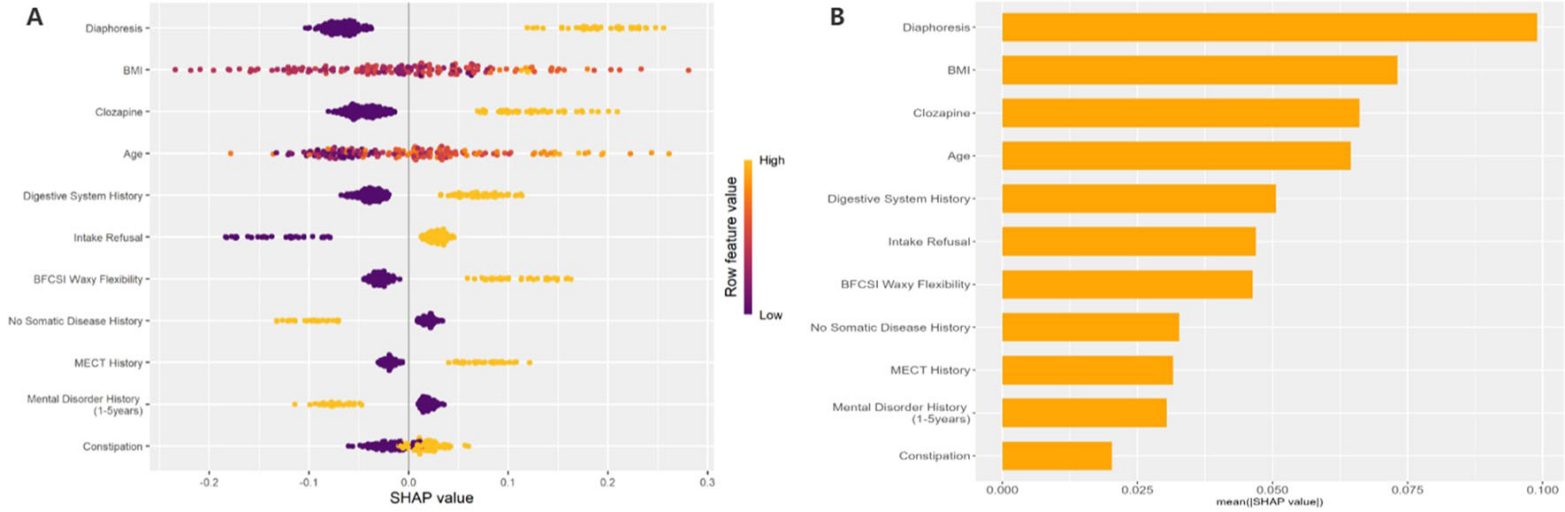
Beeswarm Plot: This figure shows the SHAP value distribution based on the GBM model, evaluating the impact of each feature on the model's predictions. The horizontal axis represents SHAP values, where larger values indicate a positive contribution to the prediction, and smaller values indicate a negative contribution. The vertical axis lists the feature names. The color of the points represents the magnitude of the feature values, with yellow indicating high values and purple indicating low values. **(B)** Feature Importance Bar Plot: This figure shows the feature importance ranking based on the mean absolute SHAP values. The horizontal axis represents the mean absolute SHAP value (mean(|SHAP value|)), which reflects the overall contribution of each feature to the model's predictions.

**Figure 5:**
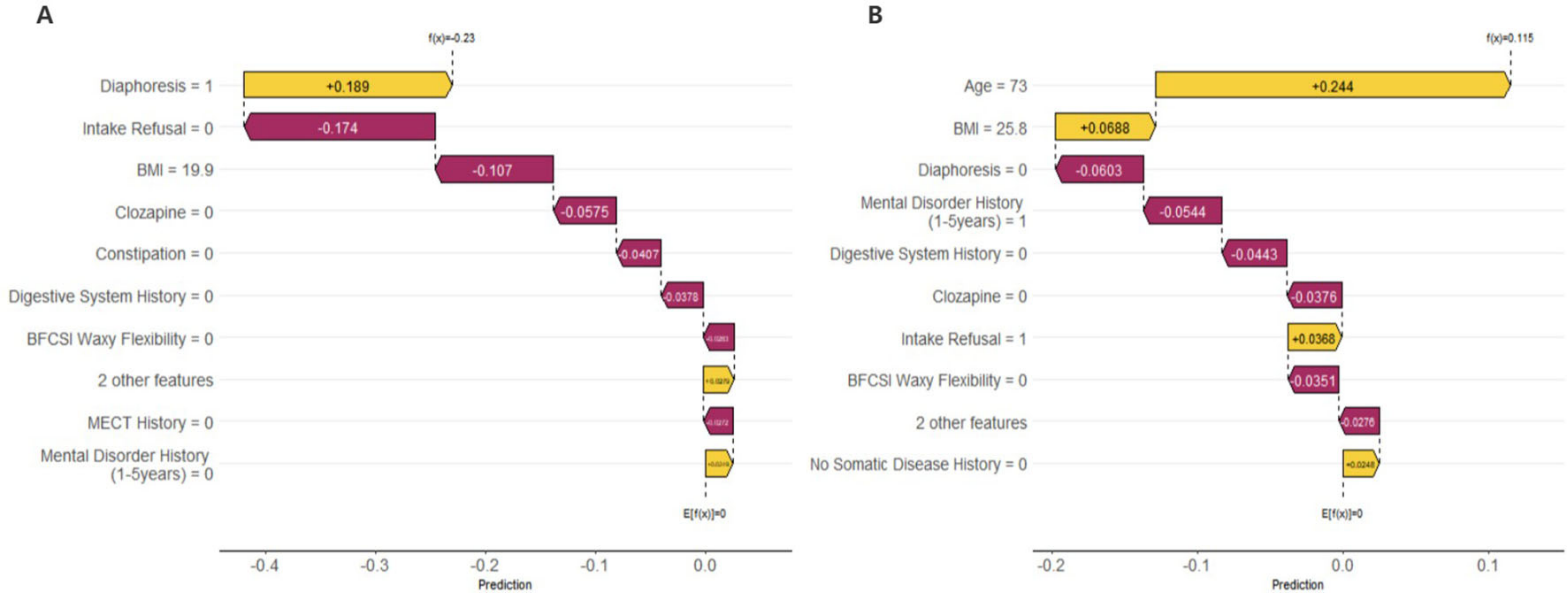
Waterfall Plot: These figures illustrate the prediction explanations for each individual sample using the GBM model. **(A)** The First Sample: Diaphoresis (Diaphoresis = 1) had a positive contribution to the model prediction (SHAP value = +0.189), whereas Intake Refusal (Intake Refusal = 0) and BMI = 19.9 had negative contributions (SHAP values = -0.174 and -0.107, respectively), indicating that these factors reduced the predicted risk for this individual. **(B)** The Last Sample: Age (Age = 73) had the highest positive contribution (SHAP value = +0.244), suggesting that advanced age was the primary predictor of pneumonia risk in this patient. Additionally, BMI = 25.8 had a mild positive contribution, while Diaphoresis (Diaphoresis = 0), Mental Disorder History (1–5 years) = 1, and Digestive System History = 0 all had negative contributions, indicating their potential protective effects in this case. These SHAP waterfall plots provide individualized insights into the factors influencing pneumonia risk predictions, enhancing the interpretability of the GBM model.

The interpretability of the GBM model based on SHAP analysis was detailed in the Beeswarm Plot, Feature Importance Bar Plot, and Waterfall Plot (**Figures 4**, **5**). The Beeswarm Plot illustrated the distribution of SHAP values for each feature. The results indicated that diaphoresis, BMI, clozapine, and age exhibited higher SHAP values, suggesting that these variables had a significant impact on the model’s predictions. Specifically, patients with a higher BMI and those using clozapine tended to contribute more positively to the model’s predictions, while diaphoresis also showed a notable effect in certain individuals (**Figure 4A**). The Feature Importance Bar Plot further highlighted that diaphoresis was the most influential predictor, followed by BMI, clozapine use, age, and digestive system history (**Figure 4B**). The Waterfall Plot visually illustrated the explanation of predictions for individual samples using the GBM model. The horizontal axis represented the cumulative SHAP values, indicating both the direction and magnitude of each feature’s contribution to the final prediction. The plot started from the model’s baseline prediction value (E[f(x)]), and SHAP values of individual features were sequentially added or subtracted, ultimately arriving at the final prediction value f(x). This visualization provided an intuitive understanding of how each feature influenced the model’s decision-making process for a given sample (**Figures 5A, B**).

**Figure 6:**
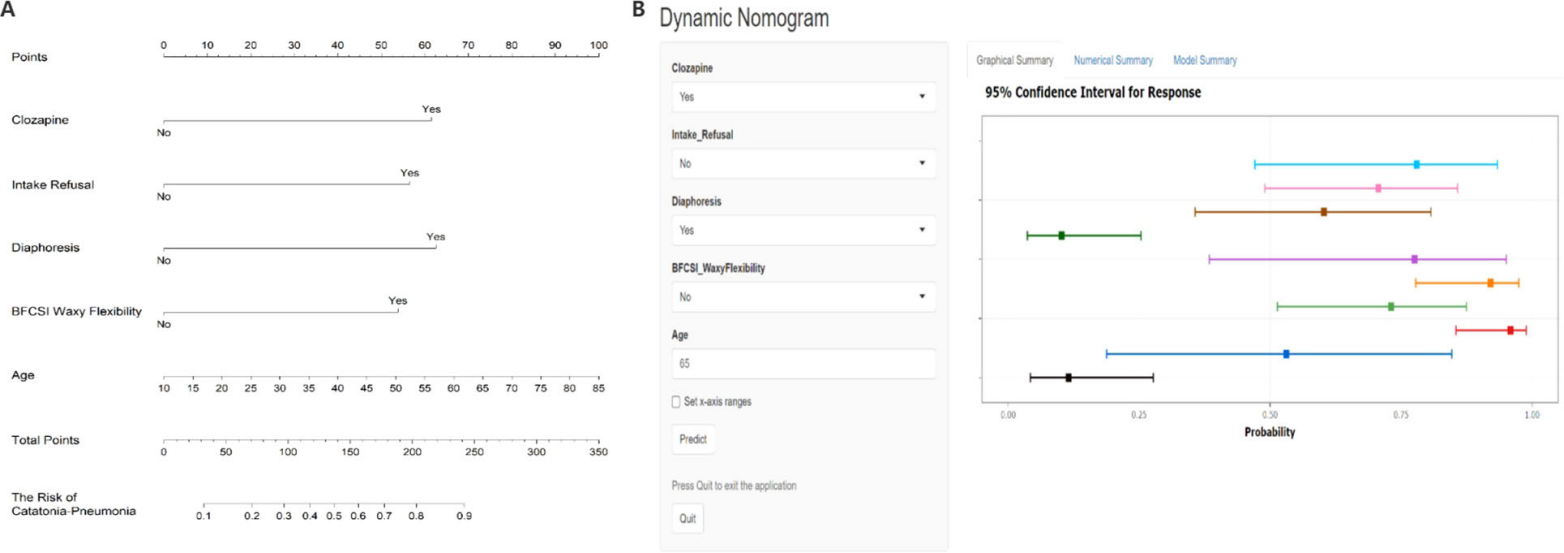
**(A)** Nomogram for predicting Catatonia-Pneumonia risk and its algorithm. First, a point was found for each variable of a Catatonia patient on the uppermost rule; then all scores were added together and the total number of points were collected. Finally, the corresponding predicted probability of Catatonia-Pneumonia was found on the lowest rule. **(B)** Dynamic Nomogram for Predicting Pneumonia Risk in Catatonia Patients. Users can input patient-specific information through dropdown menus and text fields on the left panel. Upon clicking the "Predict" button, the model generates a probability estimate with a 95% Confidence Interval (CI) for pneumonia risk, as displayed in the graphical summary on the right. The horizontal colored bars represent the predicted probabilities for different input combinations, with the squares indicating point estimates and the error bars denoting the 95% CI. A higher probability towards the right end of the x-axis indicates a greater risk of pneumonia. The "Set x-axis ranges" option allows customization of the probability scale, and model details can be accessed via the tabs at the top (Graphical Summary, Numerical Summary, Model Summary). This tool deployed via R-Shiny at https://catatonia-pneumonia.shinyapps.io/DynamicNomogram/.

**Figure 7:**
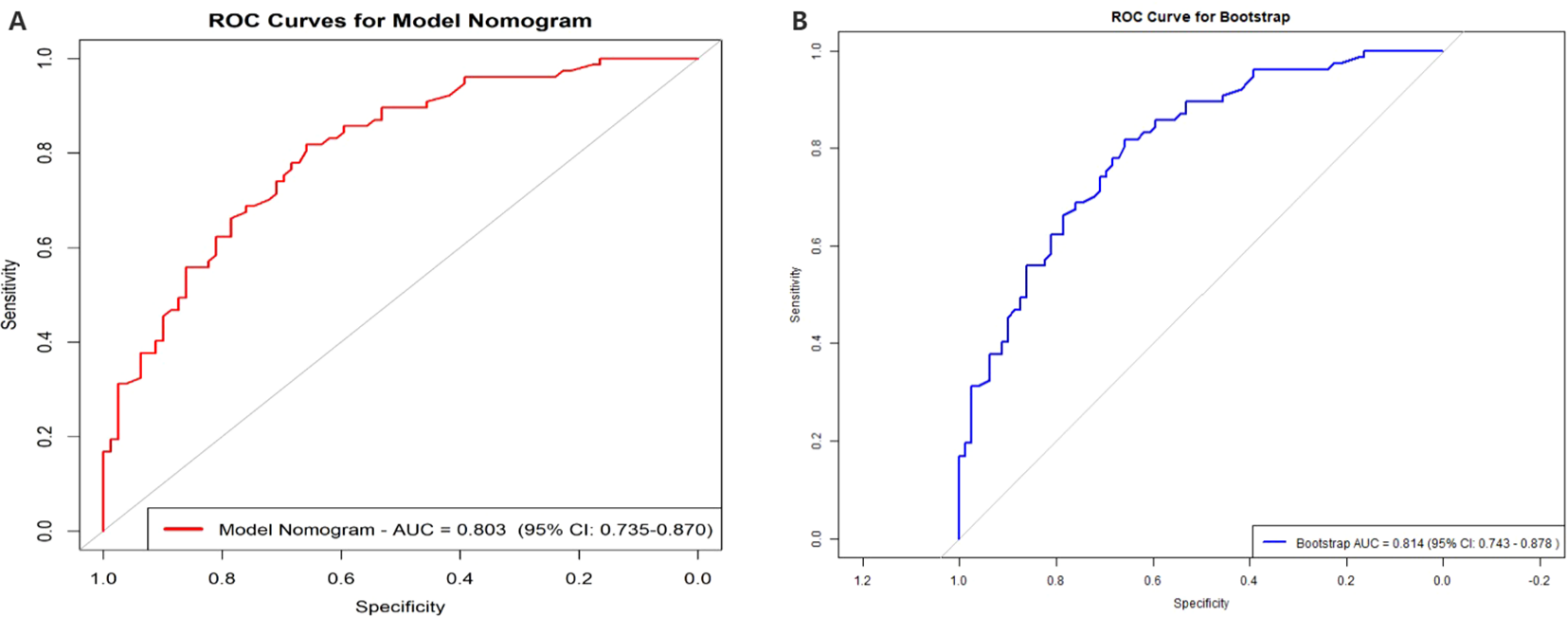
The AUC of the Model Nomogram and the internal validation. **(A)** shows the AUC of the predictive Model Nomogram, and **(B)** shows the AUC of the internal validation using the bootstrap method (resampling = 500). AUC, area under the curve.

**Figure 8:**
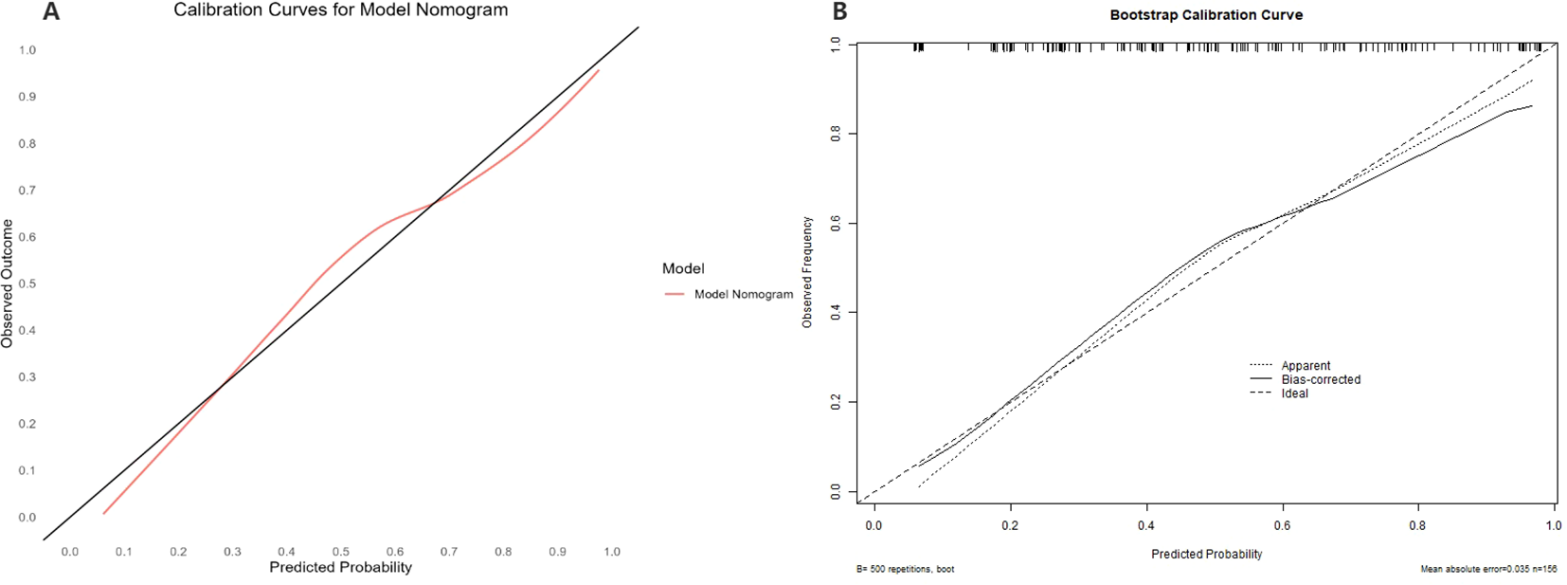
The Calibration curve of the Model Nomogram and the internal validation. **(A)** Calibration curve of the predictive model showing the degree of consistency between the predicted probability and observed probability (the Hosmer-Lemeshow test, P-value=0.5258161 (> 0.05), suggesting that it is of goodness-of-fit). The black solid line represents a perfect prediction by an ideal model, and the solid red line shows the performance of the model. **(B)** shows the Calibration curve of the internal validation using the bootstrap method (resampling = 500).

**Figure 9:**
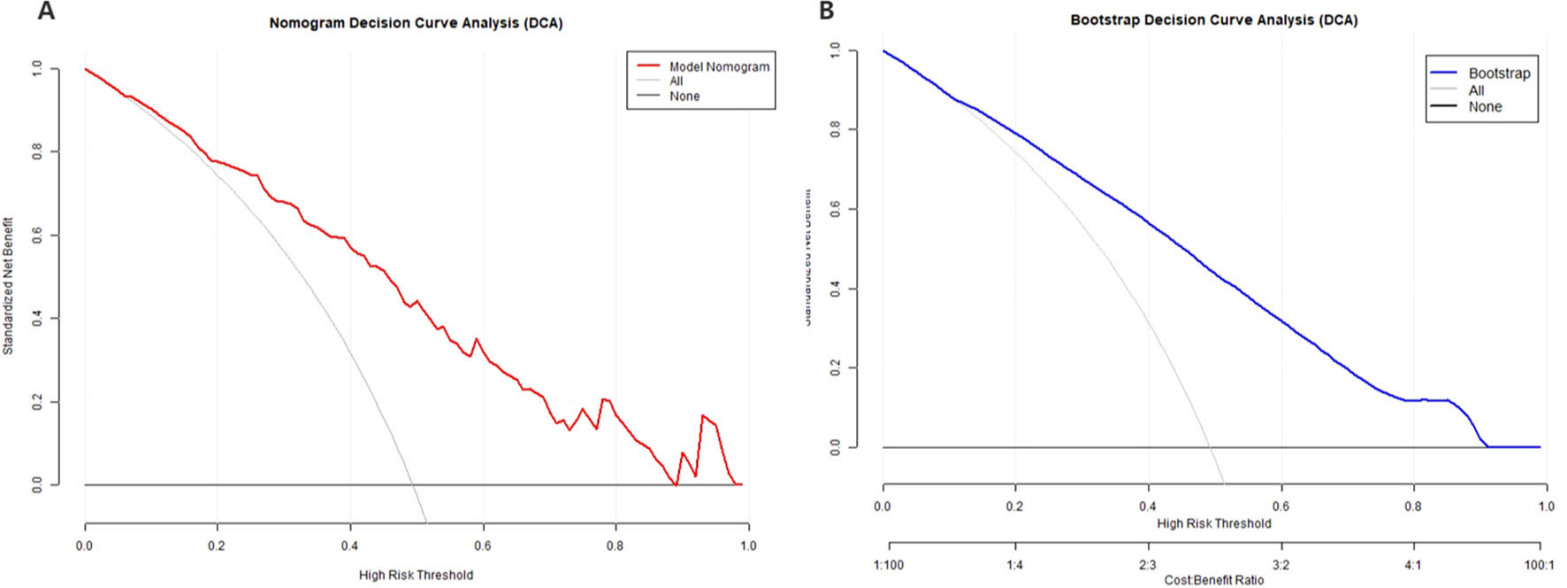
Decision Curve Analysis (DCA) of the Model Nomogram and the internal validation. **(A)** The red solid line represents the Model Nomogram. The decision curve indicates that application of this nomogram would add a net benefit when compared with either the strategy where all patients are predicted to be high-risk or the strategy where all patients are predicted to be low-risk. **(B)** Shows the Calibration curve of the internal validation using the bootstrap method (resampling = 500). DCA: Decision Curve Analysis.

A multivariate logistic regression analysis was performed on the 11 variables, ultimately selecting five variables for inclusion in the Nomogram Model: Age, Clozapine, Intake Refusal, Diaphoresis, and BFCSI Waxy Flexibility (**Tables 2**, **Figure 6A**). Model validation results were as follows: An AUC of 0.803 (95% CI: 0.735-0.870), indicating good discriminatory ability. Calibration curves and decision curve analysis demonstrated strong clinical utility of the logistic regression model. Internal validation using the bootstrap method (500 resamples) resulted in an AUC of 0.814 (95% CI: 0.743-0.878), with a Hosmer-Lemeshow test P-value of 0.5258161 (*P* > 0.05), further confirming good calibration and model fit (**Figures 7**–**9**). The final variables included in the model were tested for multicollinearity using VIF (all <10), indicating no serious multicollinearity issues. Additionally, the number of variables satisfied the 10 EPV standard, ensuring robustness and reliability of the model. In conclusion, this study developed a nomogram model based on rigorous variable selection and statistical validation, demonstrating good predictive performance and clinical applicability. A dynamic version of the nomogram for predicting pneumonia risk in catatonia patients has been deployed via R-Shiny at https://catatonia-pneumonia.shinyapps.io/DynamicNomogram/ (**Figure 6B**).

## Discussion

This study conducted a comprehensive retrospective analysis to investigate the risk of pneumonia in patients with catatonia after hospitalization. In this study, the pre-admission phase referred to the period when patients were still in the community or during their initial evaluation by general practitioners or psychiatrists at outpatient or emergency departments, aiming to predict the risk of developing pneumonia after admission. At this stage, catatonic patients often presented with rigidity and resistance, making it challenging to cooperate with psychiatric and physical examinations. Consequently, the early diagnosis of catatonia was difficult. Similarly, diagnosing pneumonia was equally challenging, especially in patients without typical symptoms such as cough or sputum production, as they might not have been able to undergo detailed investigations like chest CT or blood tests. Therefore, this study aimed to predict the risk of pneumonia at an early stage before comprehensive examinations had been conducted rather than evaluating already diagnosed cases of pneumonia. The potential risk factors collected in this study were derived from patients’ basic information, medical history, maintenance medications, outpatient evaluations, and symptoms related to catatonia. This approach allowed clinicians to quickly assess the risk of pneumonia, enabling timely risk exclusion or implementation of preventive measures. This study developed a nomogram prediction model based on five key risk factors: age, clozapine use, diaphoresis, intake refusal, and waxy flexibility. The model effectively supported risk stratification, quantitative assessment, and clinical decision-making, demonstrated strong predictive performance for pneumonia risk in hospitalized catatonia patients, and provided valuable guidance for early intervention.

In Terms of Age and Nutritional Status, This study’s model showed that older patients had a significantly higher risk of developing pneumonia, primarily due to the decline in immune system function and reduced infection resistance (24). Elderly patients often exhibited decreased mobility and prolonged bed rest during hospitalization. These factors, combined with the common symptoms of catatonia, such as mutism, immobility, and muscle rigidity, further increased the risk of pneumonia. In addition, both elderly and catatonic patients frequently experienced weakened swallowing reflexes, which significantly raised the risk of aspiration pneumonia (25–27). Compared to younger individuals, elderly patients generally had poorer nutritional status, which was also an important risk factor for pneumonia (28). Therefore, for elderly patients with catatonia, clinicians should perform comprehensive pre-admission evaluations, including chest CT, complete blood count, C-reactive protein (CRP), procalcitonin (PCT), and blood urea nitrogen (BUN) to assess infection indicators. After admission, close monitoring for symptoms such as coughing, sputum production, choking on fluids, and weakened cough reflexes is essential to enable timely interventions. Furthermore, intake refusal (refusal of food and water) before admission indicated poor short-term nutritional status, which was consistent with previous studies. A retrospective study from China on risk factors for pneumonia in patients with catatonia indicated that prolonged bed rest and smoking significantly increased the risk of pneumonia, whereas better nutritional status, such as higher albumin levels, helped reduce the risk (12). Poor nutritional status could lead to decreased immunity, indirectly increasing the risk of pneumonia (24). In the machine learning analysis phase of this study, patients with higher BMI were found to have a greater risk of pneumonia, particularly those with a BMI over 24. Although BMI was not ultimately included in the nomogram model, this finding was consistent with previous studies. Prior research suggested that BMI significantly increased the risk of pneumonia (29), especially in obese patients with reduced muscle mass who had schizophrenia. The combined effects of reduced muscle mass and increased body fat substantially elevated the risk of pneumonia in patients with stable schizophrenia. This might be related to weakened immune function and impaired sputum clearance in these patients (30). Sarcopenia has been identified as an important predictor of pneumonia in patients with stable schizophrenia (31). Therefore, obese psychiatric patients, who may appear to have an excess nutritional status, in fact, tend to have poor nutritional quality and are at risk of hidden malnutrition.

Regarding catatonia symptoms, this study identified waxy flexibility, refusal of food and water, and diaphoresis as significant risk factors for pneumonia. Waxy flexibility is a typical motor disturbance in catatonia, characterized by limb rigidity and passive resistance, with the “air pillow” phenomenon being a hallmark feature (32, 33). This symptom limits patient mobility and often leads to prolonged confinement to bed, substantially increasing the risk of respiratory tract infections. In a catatonic state, patients may experience tension in the oral and pharyngeal muscles, leading to reduced voluntary swallowing of saliva and food, thereby increasing the risk of aspiration (26). Additionally, diminished voluntary coughing and impaired sputum clearance, coupled with increased muscle tension in the pharynx, respiratory muscles, and diaphragm, may weaken the cough reflex, further heightening the risk of pneumonia. Patients with catatonia often exhibit autonomic dysfunction, characterized by tachycardia, increased respiratory rate, and profuse sweating (34). These symptoms, along with prolonged catatonic states, ultimately lead to severe dehydration (34), which, when compounded by refusal of food and water, may further contribute to the onset or worsening of pneumonia. Although heart rates exceeding 120 beats per minute and respiratory rates above 20 breaths per minute were not ultimately included in the model, univariate analysis indicated that abnormalities in these physiological indicators were potential risk factors for pneumonia. For patients exhibiting waxy flexibility, nursing interventions are particularly important, and their care management should differ from that of catatonia patients without waxy flexibility. These patients require additional nursing measures, such as passive limb movement and regular position changes, to promote blood circulation, support airway clearance, and prevent the accumulation of respiratory secretions and related complications, thereby effectively reducing pneumonia risk (35). For catatonia patients who exhibit waxy flexibility prior to hospitalization, placement in specialized care units or even psychiatric intensive care units (ICUs) should be considered to ensure a higher level of monitoring and intervention (35). Furthermore, for patients with autonomic dysfunction and refusal of food and water, adequate fluid resuscitation should be promptly administered to maintain water and electrolyte balance. Additionally, adequate nutritional support therapy is crucial for preventing and alleviating malnutrition, thereby further reducing the risk of pneumonia.

Regarding medication use, this study included the use of clozapine in the final nomogram model as a significant risk factor, which was consistent with previous research. Meta-analyses showed that the use of antipsychotic drugs was significantly associated with the occurrence of pneumonia. Both first-generation antipsychotics, such as chlorpromazine, and second-generation antipsychotics, such as olanzapine and clozapine, increased the risk of pneumonia (36). This was likely due to side effects of antipsychotic medications, such as sedation and dysphagia, which indirectly led to pneumonia (15, 37, 38). Previous studies did not explicitly examine the relationship between medication use and pneumonia in catatonia patients; however, most studies acknowledged that the use of clozapine increased the risk of pneumonia in patients with schizophrenia (15). Clozapine had immunosuppressive effects, which might have increased the risk of pneumonia through mechanisms such as affecting white blood cell counts and increasing susceptibility to bacterial infections (39). Previous studies also investigated the association between antipsychotic use and the progression of upper respiratory infections (URI) to pneumonia in patients with schizophrenia. It found that clozapine was the only antipsychotic drug that significantly increased the risk of pneumonia. Furthermore, higher doses of clozapine were associated with a greater risk of URI progressing to pneumonia (40). Compared to clozapine, which was the only antipsychotic drug included in the final model, benzodiazepines were not incorporated into the final model. However, in univariate analysis, the use of lorazepam showed statistical significance. Interestingly, lorazepam acted as a relative protective factor. Currently, the first-line treatment for catatonia primarily involves GABAergic drugs, especially benzodiazepines (BDZs). Lorazepam, as a first-line treatment for catatonia, can rapidly relieve symptoms, with a response rate of approximately 80%. It is typically administered at doses of 8 to 24 mg per day, and is generally well-tolerated without causing excessive sedation (41), particularly during acute episodes of catatonia (42). For chronic catatonia, lorazepam can also be used for long-term maintenance therapy (43). Lorazepam is a positive allosteric modulator of the GABA-A receptor, with a short duration of action and minimal effects on liver and kidney function, making it one of the best treatments for catatonia caused by various etiologies (44). However, pulmonary complications in catatonia may include pulmonary embolism, aspiration pneumonia, pneumothorax, bronchoalveolar leakage, central hypoventilation, respiratory failure, and delayed weaning from mechanical ventilation (8, 45, 46). The sedative and neuromuscular effects of benzodiazepines may suppress the cough reflex, exacerbate sputum retention, and lead to respiratory depression. Previous studies suggested that excessive use of lorazepam could result in oversedation, increasing the risk of aspiration and respiratory depression (47). Nevertheless, the univariate analysis in this study indicated that the use of lorazepam during the maintenance phase provided a protective effect against pneumonia in catatonia patients. A possible explanation is that long-term maintenance with lorazepam alleviated the severity of catatonic episodes, indirectly reducing the risk of pneumonia. Therefore, strategies for lorazepam use should be tailored to individual patient conditions. Considering the overall health status and potential comorbidities of patients, lorazepam should be used cautiously in elderly patients or those with respiratory diseases or other comorbidities that may exacerbate respiratory depression. For other catatonia patients, lorazepam should be actively used during the remission phase as maintenance therapy.

In this study, although some variables were not included in the final nomogram model, they demonstrated statistical significance in univariate analysis or were identified as important influencing factors during the machine learning selection process. For example, a prolonged history of mental disorders, particularly exceeding 10 years, multiple previous hospitalizations due to mental illness, especially between 3 to 10 admissions, and a history of electroconvulsive therapy (MECT), indirectly indicating multiple past episodes of catatonia, as well as prior hospitalizations for catatonia with pneumonia, were all associated with a higher risk of developing pneumonia. These findings are consistent with previous research, which has shown that nearly two-thirds of pneumonia cases are closely associated with treatment-resistant schizophrenia. Patients with a long history of illness and repeated hospitalizations often face multiple health complications, and due to frequent relapses and prolonged medication use, their immune function becomes compromised, ultimately increasing their susceptibility to pneumonia (39). Furthermore, patients with catatonia often encounter significant challenges in long-term care and health management due to the absence of an effective social support system, as the lack of family and social support makes it difficult for them to maintain their overall health. When symptoms such as fever, cough, or breathing difficulties occur, the lack of assistance may lead to delays in timely recognition and appropriate treatment. The findings of this study further support this perspective, as despite the relatively small sample size involving patients who were bedridden for extended periods or resided in long-term care units, other variables reflecting social support still highlighted its significance in influencing pneumonia risk. For example, in the machine learning analysis phase, being widowed was identified as a risk factor for pneumonia, while in the univariate analysis, delayed admission (7–30 days) also emerged as a contributing factor, collectively underscoring the critical role of social support in the occurrence of pneumonia. Additionally, constipation and a history of digestive system disorders were considered risk factors for pneumonia. The history of digestive system disorders included previous occurrences of gastrointestinal bleeding, intestinal obstruction, hepatitis, fatty liver, liver dysfunction, as well as pancreatic and gallbladder diseases. In clinical practice, patients with catatonia often exhibited gastrointestinal symptoms, such as constipation and even intestinal obstruction. Furthermore, patients frequently presented with concurrent digestive system disorders during the current course of catatonia or had a history of such conditions. These gastrointestinal symptoms were likely associated with persistent sympathetic nervous system hyperactivity in catatonic patients. Neurons regulating gastrointestinal function demonstrated tonic activity, while sustained GABAergic inhibitory synaptic input played a crucial role in modulating the activity of dorsal motor vagal neurons (DMV), thereby affecting gastrointestinal function (48). However, despite the findings of this study suggesting the relevance of digestive system history in catatonia with pneumonia, previous literature had paid limited attention to this issue, with only a few reported cases of catatonia complicated by gastrointestinal diseases, such as intestinal obstruction (49). Therefore, future research should further explore the potential role of digestive system diseases and history in catatonia and pneumonia, as well as the complex interplay between these factors in affected patients.

The model enabled early identification of high-risk pneumonia patients pre-admission, facilitated individualized treatment and targeted interventions for refined management. In resource-limited settings, it helped prioritize high-risk patients, optimized monitoring and care allocation to reduce pneumonia incidence, enhance healthcare quality, and shorten hospital stays. Additionally, in long-term community management, it supported regular health assessments, enabled timely risk detection, and provided continuous, personalized interventions.

## Limitations

First, the sample size of this study was relatively small. Although catatonia was frequently encountered in clinical practice, its overall incidence remained low. Therefore, this study was a single-center retrospective observational study, collecting a total of 156 patients over the past five years, reflecting the limitation of the sample size. Secondly, since the sample was derived from medical records, the high underdiagnosis rate of catatonia further restricted the sample size. Despite the redefinition of catatonia by DSM-5 and ICD-11, some clinicians still adhered to the traditional notion of catatonia as catatonic schizophrenia, leading to underdiagnosis. This may have explained why the number of catatonia cases with pneumonia was approximately equal to those without pneumonia, suggesting that milder cases may have been overlooked by clinicians. In the future, training on the diagnosis of catatonia, particularly the use of the BFCSI scale, should be strengthened to improve the diagnostic rate and thus increase the sample size. Additionally, since this study was retrospective in design, the data mainly came from past medical records, which might have been subject to information bias and incomplete records, with certain potential variables not included in the analysis. Therefore, future studies should consider more comprehensive data collection to reduce the impact of information bias. Although internal validation demonstrated good predictive ability, due to the small sample size, there was a lack of external validation using independent data. Future studies should focus on validate the model with independent data from different regions and populations. External validation could further assess the model’s applicability and reliability in different clinical settings, ensuring its value for broader application in practice. There has been a lack of clinical randomized controlled trials, case-control studies, and cohort studies. Although this study was a retrospective observational study with certain limitations, it still reflected real-world clinical conditions and provided valuable insights for future research.

## Conclusion

This study successfully developed and validated a machine learning-based nomogram model for predicting pneumonia risk in hospitalized patients with catatonia. By leveraging demographic, medical history, and clinical characteristics, five key predictive factors: Age, Clozapine, Diaphoresis, Intake Refusal, and Waxy Flexibility were identified and incorporated into the model. The nomogram demonstrated excellent predictive performance, with strong discrimination, calibration, and clinical utility, as confirmed through internal validation. This model provides a practical tool to assist clinicians in early risk assessment and personalized management of pneumonia in catatonia patients, potentially improving clinical outcomes and resource allocation.

## Data Availability

The authors intend to share all individual deidentified participant data except patients’privacy. Further inquiries can be directed to the corresponding author.

## Supplementary material

Supplementary Material 1 The patient dataset comprised 213 variables. Univariate analyses were conducted using T-tests, U-tests, and chi-square tests, identifying 46 variables with P-values less than 0.05, as detailed in the Supplementary Tables.

Supplementary Material 2 TRIPOD (Transparent Reporting of a Multivariable Prediction Model for Individual Prognosis or Diagnosis) Checklist for Model Development and Validation.
