# Supplementary material for: Construction and validation of a machine learning-based nomogram model for predicting pneumonia risk in patients with catatonia: a retrospective observational study
Source: Front Psychiatry. 2025 Mar 14;16:1557659. doi: 10.3389/fpsyt.2025.1557659 (PMC11951867; doi:10.3389/fpsyt.2025.1557659)
Supplement: Supplementary Material 1 — The patient dataset comprised 213 variables. Univariate analyses were conducted using T-tests, U-tests, and chi-square tests, identifying 46 variables with P-values less than 0.05, as detailed in the Supplementary Tables. [file SupplementaryFile1.doc]

| **Supplementary Table 1 Demographic Characteristics** | | | | |
| --- | --- | --- | --- | --- |
| **Variables** | **Total** | **No-PNA Group** | **PNA Group** | ***P*-value** |
| Gender (N(%)) |  |  |  |  |
| Male | 66 (42.31%) | 33 (41.77%) | 33 (42.86%) | 0.891 |
| Female | 90 (57.69%) | 46 (58.23%) | 44 (57.14%) | 0.891 |
| Age (M[Q1, Q3]) | 48 (32, 62) | 40 (21, 57) | 53 (39, 65) | 0.001* |
| <18 (N(%)) | 13 (8.33%) | 11 (13.92%) | 2 (2.60%) | 0.022* |
| 18-34 (N(%)) | 33 (21.15%) | 21 (26.58%) | 12 (15.58%) | 0.096 |
| 35-60 (N(%)) | 67 (42.95%) | 31 (39.24%) | 36 (46.75%) | 0.344 |
| ＞60 (N(%)) | 43 (27.56%) | 16 (20.25%) | 27 (35.06%) | 0.040* |
| Height (M±SD) | 163.01 ± 7.64 | 163.67 ± 7.57 | 162.34 ± 7.71 | 0.276 |
| Weight (M[Q1, Q3]) | 60.25 (52, 70) | 60 (50.75, 66.25) | 63 (53, 73) | 0.086 |
| BMI (M[Q1, Q3]) | 22.88 (20.31, 25.61) | 22.15 (19.78, 24.67) | 24.19 (21.03, 27.04) | 0.020* |
| <18 (N(%)) | 13 (8.33%) | 9 (11.39%) | 4 (5.19%) | 0.172 |
| 18-24 (N(%)) | 78 (50%) | 44 (55.70%) | 34 (44.16%) | 0.150 |
| >24 (N(%)) | 65 (41.67%) | 26 (32.91%) | 39 (50.65%) | 0.026* |
| Marital (N(%)) |  |  |  |  |
| Single (Age ＜18 Excluded) | 37 (23.72%) | 21 (26.58%) | 16 (20.78%) | 0.395 |
| Married | 87 (55.77%) | 45 (56.96%) | 42 (54.55%) | 0.761 |
| Divorced | 9 (5.77%) | 2 (2.53%) | 7 (9.09%) | 0.100 |
| Widowed | 10 (6.41%) | 0 (0.00%) | 10 (12.99%) | 0.989 |
| Educational level (N(%)) |  |  |  |  |
| Illiteracy | 16 (10.26%) | 8 (10.13%) | 8 (10.39%) | 0.957 |
| Primary School | 33 (21.15%) | 14 (17.72%) | 19 (24.68%) | 0.289 |
| Middle School | 47 (30.13%) | 24 (30.38%) | 23 (29.87%) | 0.945 |
| High School | 32 (20.51%) | 15 (18.99%) | 17 (22.08%) | 0.633 |
| University | 28 (17.95%) | 18 (22.78%) | 10 (12.99%) | 0.115 |
| Educational and Employment Status (N(%)) |  |  |  |  |
| Enrollment | 10 (6.41%) | 7 (8.86%) | 3 (3.90%) | 0.218 |
| Dropout | 11 (7.05%) | 10 (12.66%) | 1 (1.30%) | 0.024* |
| Unemployment | 53 (33.97%) | 26 (32.91%) | 27 (35.06%) | 0.776 |
| Employment | 24 (15.38%) | 13 (16.46%) | 11 (14.29%) | 0.707 |
| Retirement | 31 (19.87%) | 13 (16.46%) | 18 (23.38%) | 0.281 |
| Peasant | 27 (17.31%) | 10 (12.66%) | 17 (22.08%) | 0.124 |
| Abbreviations: BMI, Body Mass Index  **P*＜0.05 | | | | |

| **Supplementary Table 2 Social Support and Substance Dependence** | | | | |
| --- | --- | --- | --- | --- |
| **Variables** | **Total** | **No-PNA Group** | **PNA Group** | ***P*-value** |
| **Social Support (N(%))** | | | | |
| Parental Survival Status (Age >60 Excluded) |  |  |  |  |
| Both Parents Deceased | 5 (3.21%) | 1 (1.27%) | 4 (5.19%) | 0.199 |
| One Parent Deceased | 11 (7.05%) | 7 (8.86%) | 4 (5.19%) | 0.377 |
| Both Parents Alive | 98 (62.82%) | 55 (69.62%) | 43 (55.84%) | 0.076 |
| Living With Parents (Age ＜18 Excluded) | 32 (20.51%) | 18 (22.79%) | 14 (18.18%) | 0.477 |
| Reproductive Status (Age ＜35 Excluded) |  |  |  |  |
| Childless | 18 (11.54%) | 5 (6.33%) | 13 (16.88%) | 0.047* |
| One Child | 48 (30.77%) | 21 (26.58%) | 27 (35.06%) | 0.252 |
| Two or More Children | 42 (26.92%) | 20 (25.32%) | 22 (28.57%) | 0.647 |
| Living With Children (Age ＜35 Excluded) | 36 (23.08%) | 17 (21.52%) | 19 (24.68%) | 0.640 |
| Solitary Living | 22 (14.1%) | 8 (10.13%) | 14 (18.18%) | 0.153 |
| Guardian and Accompanying Person |  |  |  |  |
| Spouse | 46 (29.49%) | 28 (35.44%) | 18 (23.38%) | 0.100 |
| Parents or Offsprings | 81 (51.92%) | 39 (49.37%) | 42 (54.55%) | 0.518 |
| Siblings | 25 (16.03%) | 9 (11.39%) | 16 (20.78%) | 0.115 |
| Extended Family | 4 (2.56%) | 3 (3.80%) | 1 (1.30%) | 0.346 |
| Bedridden Status | 12 (7.69%) | 3 (3.80%) | 9 (11.69%) | 0.078 |
| Long Term Care Facility | 8 (5.13%) | 1 (1.27%) | 7 (9.09%) | 0.058 |
| Rural Residency | 78 (50%) | 40 (50.63%) | 38 (49.35%) | 0.873 |
| Medical Insurance Status | 134 (85.9%) | 63 (79.75%) | 71 (92.21%) | 0.031* |
| **Substance Dependence (N(%))** | | | | |
| Past Smoking | 16 (10.26%) | 8 (10.13%) | 8 (10.39%) | 0.957 |
| Current Smoking | 9 (5.77%) | 4 (5.06%) | 5 (6.49%) | 0.702 |
| Past Drinking | 18 (11.54%) | 11 (13.92%) | 7 (9.09%) | 0.348 |
| Current Drinking | 11 (7.05%) | 5 (6.33%) | 6 (7.79%) | 0.722 |
| **P*＜0.05 | | | | |

| **Supplementary Table 3 Previous History of Somatic Disorders** | | | | |
| --- | --- | --- | --- | --- |
| **Variables (N(%))** | **Total** | **No-PNA Group** | **PNA Group** | ***P*-value** |
| No Somatic Disease History | 26 (16.67%) | 20 (25.32%) | 6 (7.79%) | 0.005* |
| Cardiovascular History | 45 (28.85%) | 17 (21.52%) | 28 (36.36%) | 0.043* |
| Arrhythmia | 5 (3.21%) | 0 (0.00%) | 5 (6.49%) | 0.988 |
| Coronary Disease | 16 (10.26%) | 6 (7.59%) | 10 (12.99%) | 0.272 |
| Hypertension | 36 (23.08%) | 12 (15.19%) | 24 (31.17%) | 0.020* |
| Respiratory History | 5 (3.21%) | 3 (3.80%) | 2 (2.60%) | 0.672 |
| Tracheitis | 2 (1.28%) | 1 (1.27%) | 1 (1.30%) | 0.985 |
| Obstructive Pulmonary Disease | 2 (1.28%) | 2 (2.53%) | 0 (0.00%) | 0.988 |
| Pulmonary Embolism | 1 (0.64%) | 0 (0.00%) | 1 (1.30%) | 0.987 |
| Digestive System History | 55 (35.26%) | 19 (24.05%) | 36 (46.75%) | 0.003* |
| Bleeding | 8 (5.13%) | 1 (1.27%) | 7 (9.09%) | 0.058 |
| Obstruction | 7 (4.49%) | 1 (1.27%) | 6 (7.79%) | 0.084 |
| Hepatitis | 4 (2.56%) | 1 (1.27%) | 3 (3.90%) | 0.323 |
| Fatty Liver | 17 (10.9%) | 7 (8.86%) | 10 (12.99%) | 0.411 |
| Liver Dysfunction | 18 (11.54%) | 8 (10.13%) | 10 (12.99%) | 0.577 |
| Cholecystitis | 7 (4.49%) | 2 (2.53%) | 5 (6.49%) | 0.249 |
| Pancreatitis | 1 (0.64%) | 0 (0.00%) | 1 (1.30%) | 0.987 |
| Appendicitis | 4 (2.56%) | 3 (3.80%) | 1 (1.30%) | 0.346 |
| Nephrology History | 22 (14.1%) | 8 (10.13%) | 14 (10.39%) | 0.153 |
| Renal dysfunction | 9 (5.77%) | 4 (5.06%) | 5 (6.49%) | 0.702 |
| Rhabdomyolysis | 10 (6.41%) | 4 (5.06%) | 6 (7.79%) | 0.490 |
| Hyperuricemia | 7 (4.49%) | 1 (1.27%) | 6 (7.79%) | 0.084 |
| Metabolic Disorders History | 38 (24.36%) | 16 (20.25%) | 22 (28.57%) | 0.228 |
| Diabetes | 18 (11.54%) | 6 (7.59%) | 12 (15.58%) | 0.126 |
| Thyroid | 10 (6.41%) | 4 (5.06%) | 6 (7.79%) | 0.490 |
| Hyperlipidemia | 13 (8.33%) | 6 (7.59%) | 7 (9.09%) | 0.736 |
| Epilepsy | 1 (0.64%) | 0 (0.00%) | 1 (1.30%) | 0.987 |
| Cerebrovascular | 9 (5.77%) | 3 (3.80%) | 6 (7.79%) | 0.294 |
| Anemia | 12 (7.69%) | 3 (3.80%) | 9 (11.69%) | 0.078 |
| Thrombopenia | 3 (1.92%) | 0 (0.00%) | 3 (3.90%) | 0.985 |
| Thrombosis | 10 (6.41%) | 4 (5.06%) | 6 (7.79%) | 0.490 |
| Coagulopathy | 7 (4.49%) | 5 (6.33%) | 2 (2.60%) | 0.275 |
| Tumor | 3 (1.92%) | 3 (3.80%) | 0 (0.00%) | 0.985 |
| Hypoproteinemia | 1 (0.64%) | 0 (0.00%) | 1 (1.30%) | 0.987 |
| Fractures | 8 (5.13%) | 3 (3.80%) | 5 (6.49%) | 0.450 |
| Surgery | 17 (10.9%) | 6 (7.59%) | 11 (14.29%) | 0.187 |
| **P*＜0.05 | | | | |

| **Supplementary Table 4 Previous History of Mental Disordor and Catatonia** | | | | | | | | |  |
| --- | --- | --- | --- | --- | --- | --- | --- | --- | --- |
| **Variables** | | | | | **Total** | **No-PNA Group** | **PNA Group** | ***P*-value** |  |
| **Previous History of Mental Disordor** | | | | | | | | |  |
| Family History of Mental Disordor (N(%)) | | | | | 38 (24.36%) | 17 (21.52%) | 21 (27.27%) | 0.403 |  |
| Age at Onset (*years* (M[Q1, Q3])) | | | | | 25 (19, 37) | 24 (17, 37) | 27 (20, 37) | 0.134 |  |
| <18 *years* (N(%)) | | | | | 32 (20.51%) | 22 (27.85%) | 10 (12.99%) | 0.024* |  |
| 18-34 *years* (N(%)) | | | | | 77 (49.36%) | 33 (41.77%) | 44 (57.14%) | 0.056 |  |
| 35-60 *years* (N(%)) | | | | | 30 (19.23%) | 18 (22.78%) | 12 (15.58%) | 0.256 |  |
| ＞60 *years* (N(%)) | | | | | 17 (10.9%) | 6 (7.59%) | 11 (14.29%) | 0.187 |  |
| History of Mental Disordor (*years* (M[Q1, Q3])) | | | | | 10 (1, 28.5) | 3 (0.50, 20) | 17 (4, 30) | 0.029* |  |
| First Episode (N(%)) | | | | | 23 (14.74%) | 15 (18.99%) | 8 (10.39%) | 0.135 |  |
| ＜1 *years* (N(%)) | | | | | 12 (7.69%) | 7 (8.86%) | 5 (6.49%) | 0.580 |  |
| 1-5 *years* (N(%)) | | | | | 32 (20.51%) | 24 (30.38%) | 8 (10.39%) | 0.003* |  |
| 6-10 *years* (N(%)) | | | | | 12 (7.69%) | 7 (8.86%) | 5 (6.49%) | 0.580 |  |
| 11-20 *years* (N(%)) | | | | | 27 (17.31%) | 7 (8.86%) | 20 (25.97%) | 0.007* |  |
| ＞20 *years* (N(%)) | | | | | 50 (32.05%) | 19 (24.05%) | 31 (40.26%) | 0.031* |  |
| Previous Hospitalizations due to Mental Disordor (*Number* (M[Q1, Q3])) | | | | | 1 (0, 4) | 1 (0, 3) | 3 (0, 5) | 0.044* |  |
| No Hospitalization (N(%)) | | | | | 61 (39.1%) | 38 (48.10%) | 23 (29.87%) | 0.021* |  |
| 1-2 (N(%)) | | | | | 34 (21.79%) | 20 (25.32%) | 14 (18.18%) | 0.282 |  |
| 3-10 (N(%)) | | | | | 49 (31.41%) | 17 (21.52%) | 32 (41.56%) | 0.008* |  |
| ＞10 (N(%)) | | | | | 12 (7.69%) | 4 (5.06%) | 8 (10.39%) | 0.221 |  |
| **Previous History of Catatonia** | | | | | | | | |  |
| Previous Catatonia Episodes (*Number* (M[Q1, Q3])) | | | | | 1 (0, 2) | 0 (0, 1) | 1(0, 2) | 0.106 |  |
| 0 (N(%)) | | | | | 90 (57.69%) | 52 (65.82%) | 38 (49.35%) | 0.038* |  |
| 1-2 (N(%)) | | | | | 38 (24.36%) | 17 (21.52%) | 21 (27.27%) | 0.403 |  |
| ≥3 (N(%)) | | | | | 28 (17.95%) | 10 (12.66%) | 18 (23.38%) | 0.085 |  |
| Previous Hospitalizations due to Catatonia (*Number* (M[Q1, Q3])) | | | | | 0 (0, 1) | 0 (0, 1) | 0 (0, 2) | 0.080 |  |
| No Hospitalization (N(%)) | | | | | 5 (3.21%) | 2 (2.53%) | 3 (3.90%) | 0.631 |  |
| 1-2 (N(%)) | | | | | 36 (23.08%) | 16 (20.25%) | 20 (25.97%) | 0.397 |  |
| ≥3 (N(%)) | | | | | 25 (16.03%) | 9 (11.39%) | 16 (20.78%) | 0.115 |  |
| Previous Hospitalizations due to Catatonia with Pneumonia (*Yes*) | | | | | 13 (8.33%) | 2 (2.53%) | 11 (14.29%) | 0.018* |  |
| **P*＜0.05 | | | | |  |  |  |  |  |
| **Supplementary Table 5 Maintenance Medication During Remission Phase for Mental Disorders** | | | | | | | | | |
| **Variables** | **Total** | **No-PNA Group** | **PNA Group** | ***P*-value** | | | | | |
| **APDs** | | | | | | | | | |
| APDs Dosage (M[Q1, Q3]) | 5 (0, 13.33) | 3.84 (0.00, 12.50) | 6.25 (2.50, 15.83) | 0.229 | | | | | |
| 0 (N(%)) | 44 (28.21%) | 29 (36.71%) | 15 (19.48%) | 0.018* | | | | | |
| <5 (N(%)) | 26 (16.67%) | 11 (13.92%) | 15 (19.48%) | 0.354 | | | | | |
| 5-9 (N(%)) | 32 (20.51%) | 16 (20.25%) | 16 (20.78%) | 0.935 | | | | | |
| 10-20 (N(%)) | 33 (21.15%) | 13 (16.46%) | 20 (25.97%) | 0.148 | | | | | |
| ＞20 (N(%)) | 21 (13.46%) | 10 (12.66%) | 11 (14.29%) | 0.766 | | | | | |
| **Particular APDs (N(%))** | | | | | | | | | |
| FGAs | 7 (4.49%) | 4 (5.06%) | 3 (3.90%) | 0.726 | | | | | |
| Promethazine | 3 (1.92%) | 2 (2.53%) | 1 (1.30%) | 0.582 | | | | | |
| Chlorpromazine | 1 (0.64%) | 0 (0.00%) | 1 (1.30%) | 0.987 | | | | | |
| Haloperidol | 3 (1.92%) | 2 (2.53%) | 1 (1.30%) | 0.582 | | | | | |
| SGAs | 111 (71.15%) | 49 (62.03%) | 62 (80.52%) | 0.012* | | | | | |
| Quetiapine | 26 (16.67%) | 15 (18.99%) | 11 (14.29%) | 0.432 | | | | | |
| Paliperidone | 10 (6.41%) | 5 (6.33%) | 5 (6.49%) | 0.967 | | | | | |
| Clozapine | 42 (26.92%) | 9 (11.39%) | 33 (42.86%) | ＜0.001* | | | | | |
| Olanzapine | 38 (24.36%) | 21 (26.58%) | 17 (22.08%) | 0.513 | | | | | |
| Amisulpride | 5 (3.21%) | 2 (2.53%) | 3 (3.90%) | 0.631 | | | | | |
| Sulpiride | 11 (7.05%) | 4 (5.06%) | 7 (9.09%) | 0.332 | | | | | |
| Aripiprazole | 9 (5.77%) | 6 (7.59%) | 3 (3.90%) | 0.330 | | | | | |
| Risperidone | 18 (11.54%) | 6 (7.59%) | 12 (15.58%) | 0.126 | | | | | |
| Blonanserin | 3 (1.92%) | 1 (1.27%) | 2 (2.60%) | 0.553 | | | | | |
| Longacting Injectable | 2 (1.28%) | 0 (0.00%) | 2 (2.60%) | 0.988 | | | | | |
| **BDZs** | | | | | | | | | |
| BDZs Dosage (M[Q1, Q3]) | 0 (0, 7.5) | 0 (0, 8.75) | 0 (0, 5) | 0.742 | | | | | |
| 0 (N(%)) | 110 (70.51%) | 53 (67.09%) | 57 (74.03%) | 0.343 | | | | | |
| ＜5 (N(%)) | 1 (0.64%) | 1 (1.27%) | 0 (0.00%) | 0.987 | | | | | |
| 5-9 (N(%)) | 8 (5.13%) | 5 (6.33%) | 3 (3.90%) | 0.495 | | | | | |
| 10-20 (N(%)) | 23 (14.74%) | 13 (16.46%) | 10 (12.99%) | 0.542 | | | | | |
| ＞20 (N(%)) | 14 (8.97%) | 7 (8.86%) | 7 (9.09%) | 0.960 | | | | | |
| **Particular BDZs (N(%))** | | | | | | | | | |
| Clonazepam | 7 (4.49%) | 2 (2.53%) | 5 (6.49%) | 0.249 | | | | | |
| Alprazolam | 8 (5.13%) | 6 (7.59%) | 2 (2.60%) | 0.177 | | | | | |
| Estazolam | 1 (0.64%) | 0 (0.00%) | 1 (1.30%) | 0.987 | | | | | |
| Midazolam | 2 (1.28%) | 0 (0.00%) | 2 (2.60%) | 0.988 | | | | | |
| Oxazepam | 7 (4.49%) | 5 (6.33%) | 2 (2.60%) | 0.275 | | | | | |
| Lorazepam | 21 (13.46%) | 15 (18.99%) | 6 (7.79%) | 0.047* | | | | | |
| Diazepam | 2 (1.28%) | 1 (1.27%) | 1 (1.30%) | 0.985 | | | | | |
| Eszopiclone | 6 (3.85%) | 2 (2.53%) | 4 (5.19%) | 0.397 | | | | | |
| **Mood Stabilizers (N(%))** | | | | | | | | | |
| Mood Stabilizers | 21 (13.46%) | 9 (11.39%) | 12 (15.58%) | 0.445 | | | | | |
| Lithium | 4 (2.56%) | 2 (2.53%) | 2 (2.60%) | 0.979 | | | | | |
| Valproic Acid | 17 (10.9%) | 7 (8.86%) | 10 (12.99%) | 0.411 | | | | | |
| Other Mood Stabilizers | 3 (1.92%) | 1 (1.27%) | 2 (2.60%) | 0.553 | | | | | |
| **Other Treatments (N(%))** | | | | | | | | | |
| Antidepressants History | 46 (29.49%) | 28 (35.44%) | 18 (23.38%) | 0.100 | | | | | |
| MECT History | 33 (21.15%) | 8 (10.13%) | 25 (32.47%) | 0.001* | | | | | |
| Abbreviations: APDs, Antipsychotic Drugs; GAs, First-Generation Antipsychotics; SGAs, Second-Generation Antipsychotics; BDZs, Benzodiazepines; MECT, Modified Electroconvulsive Therapy  **P*＜0.05 | | | | | | | | | |
|

| **Supplementary Table 6 Current Medical Condition** | | | | |
| --- | --- | --- | --- | --- |
| **Variables** | **Total** | **No-PNA Group** | **PNA Group** | ***P*-value** |
| **Duration of Time to Admission** | | | | |
| Emergency Admission (*Yes*) | 69 (44.23%) | 28 (35.44%) | 41 (53.25%) | 0.026* |
| Multiple Transfers Admission (*Yes*) | 82 (52.56%) | 43 (54.43%) | 39 (50.65%) | 0.636 |
| Cross Regional Medical Settlement (*Yes*) | 82 (52.56%) | 47 (59.49%) | 35 (45.45%) | 0.080 |
| Time to Admission *days* (M[Q1, Q3]) | 8.5 (5, 28.25) | 8 (4, 30) | 10 (5, 22) | 0.498 |
| ＜7 (N(%)) | 67 (42.95%) | 38 (48.10%) | 29 (37.66%) | 0.189 |
| 7-30 (N(%)) | 51 (32.69%) | 19 (24.05%) | 32 (41.56%) | 0.021* |
| ＞30 (N(%)) | 38 (24.36%) | 22 (27.85%) | 16 (20.78%) | 0.305 |
| **Trigger Factors (N(%))** | | | | |
| Drug Overdose (*Yes*) | 9 (5.77%) | 2 (2.53%) | 7 (9.09%) | 0.100 |
| Drug Discontinuation (*Yes*) | 46 (29.49%) | 23 (29.11%) | 23 (29.87%) | 0.918 |
| Stressful Events (*Yes*) | 27 (17.31%) | 17 (21.52%) | 10 (12.99%) | 0.163 |
| **Baseline Vital Signs** | | | | |
| HP (M±SD) | 98.54 ± 19.09 | 94.59 ± 17.44 | 102.58 ± 19.95 | 0.010* |
| 60-100 (N(%)) | 97 (62.18%) | 56 (70.89%) | 41 (53.25%) | 0.024* |
| 100-120 (N(%)) | 34 (21.79%) | 17 (21.52%) | 17 (22.08%) | 0.933 |
| ＞120 (N(%)) | 25 (16.03%) | 6 (7.59%) | 19 (24.68%) | 0.006* |
| RR (M[Q1, Q3]) | 18 (18, 18) | 18 (18, 18) | 18 (18, 20) | 0.028* |
| 12-20 (N(%)) | 141 (90.38%) | 77 (97.47%) | 64 (83.12%) | 0.008* |
| ＞20 (N(%)) | 15 (9.62%) | 2 (2.53%) | 13 (16.88%) | 0.008* |
| SBP (M±SD) | 135.08 ± 19.58 | 132.85 ± 19.43 | 137.38 ± 19.59 | 0.150 |
| ＜90 (N(%)) | 1 (0.64%) | 0 (0.00%) | 1 (1.30%) | 0.123 |
| 90-140 (N(%)) | 1 (0.64%) | 57 (72.15%) | 46 (59.74%) | 0.987 |
| ＞140 (N(%)) | 103 (66.03%) | 22 (27.85%) | 30 (38.96%) | 0.103 |
| DBP (M±SD) | 84.98 ± 11.62 | 83.56 ± 10.53 | 86.44 ± 12.55 | 0.142 |
| ＜60 (N(%)) | 2 (1.28%) | 1 (1.27%) | 1 (1.30%) | 0.985 |
| 60-90 (N(%)) | 117 (75%) | 65 (82.28%) | 52 (67.53%) | 0.036* |
| ＞90 (N(%)) | 37 (23.72%) | 13 (16.46%) | 24 (31.17%) | 0.033* |
| **General Somatic Symptoms (N(%))** | | | | |
| Intake Refusal | 128 (82.05%) | 58 (73.42%) | 70 (90.91%) | 0.006* |
| Urinary Retention | 33 (21.15%) | 12 (15.19%) | 21 (27.27%) | 0.068 |
| Urinary Incontinence | 25 (16.03%) | 10 (12.66%) | 15 (19.48%) | 0.249 |
| Constipation | 85 (54.49%) | 33 (41.77%) | 52 (67.53%) | 0.001* |
| Fecal Incontinence | 9 (5.77%) | 5 (6.33%) | 4 (5.19%) | 0.762 |
| Vomiting | 7 (4.49%) | 1 (1.27%) | 6 (7.79%) | 0.084 |
| Diaphoresis | 40 (25.64%) | 9 (11.39%) | 31 (40.26%) | ＜0.001* |
| Pressure Ulcer | 10 (6.41%) | 3 (3.80%) | 7 (9.09%) | 0.190 |
| Weight Loss | 38 (24.36%) | 16 (20.25%) | 22 (28.57%) | 0.228 |
| Weight Gain | 1 (0.64%) | 1 (1.27%) | 0 (0.00%) | 0.987 |
| Abbreviations: HP, Heart Rate; RR, Respiratory Rate; SBP, Systolic Blood Pressure; DBP, Diastolic Blood Pressure  **P*＜0.05 | | | | |
|

| **Supplementary Table 7 The Diagnosis and Symptoms of Catatonia** | | | | |
| --- | --- | --- | --- | --- |
| **Variables** | **Total** | **No-PNA Group** | **PNA Group** | ***P*-value** |
| **Diagnosis (N(%))** | | | | |
| Autism Spectrum Disorder | 8 (5.13%) | 5 (6.33%) | 3 (3.90%) | 0.495 |
| Schizophrenia | 65 (41.67%) | 30 (37.97%) | 35 (45.45%) | 0.344 |
| Acute Transient Psychotic Disorder | 9 (5.77%) | 6 (7.59%) | 3 (3.90%) | 0.330 |
| Bipolar Disorder | 17 (10.9%) | 7 (8.86%) | 10 (12.99%) | 0.411 |
| Major Depressive Disorder | 29 (18.59%) | 15 (18.99%) | 14 (18.18%) | 0.897 |
| DissociativeDisorders | 12 (7.69%) | 7 (8.86%) | 5 (6.49%) | 0.580 |
| Obsessive Compulsive Disorder | 1 (0.64%) | 1 (1.27%) | 0 (0.00%) | 0.987 |
| Dementia | 4 (2.56%) | 0 (0.00%) | 4 (5.19%) | 0.989 |
| Vascular Dementia | 6 (3.85%) | 4 (5.06%) | 2 (2.60%) | 0.432 |
| Alcohol Induced Mental Disorder | 3 (1.92%) | 2 (2.53%) | 1 (1.30%) | 0.582 |
| Stress Disorders | 2 (1.28%) | 2 (2.53%) | 0 (0.00%) | 0.988 |
| **BFCRI (N(%))** | | | | |
| Excitement | 49 (31.41%) | 27 (34.18%) | 22 (28.57%) | 0.451 |
| Stupor | 129 (82.69%) | 61 (77.22%) | 68 (88.31%) | 0.071 |
| Mutism | 127 (81.41%) | 60 (75.95%) | 67 (87.01%) | 0.080 |
| Staring | 102 (65.38%) | 44 (55.70%) | 58 (75.32%) | 0.011* |
| Posturing | 138 (88.46%) | 70 (88.61%) | 68 (88.31%) | 0.954 |
| Grimacing | 29 (18.59%) | 21 (26.58%) | 8 (10.39%) | 0.012* |
| Echopraxia | 13 (8.33%) | 11 (13.92%) | 2 (2.60%) | 0.022* |
| Stereotypy | 49 (31.41%) | 29 (36.71%) | 20 (25.97%) | 0.150 |
| Mannerisms | 48 (30.77%) | 29 (36.71%) | 19 (24.68%) | 0.105 |
| Verbigeration | 32 (20.51%) | 20 (25.32%) | 12 (15.58%) | 0.135 |
| Rigidity | 135 (86.54%) | 62 (78.48%) | 73 (94.81%) | 0.006* |
| Negativism | 134 (85.9%) | 67 (84.81%) | 67 (87.01%) | 0.693 |
| Waxy Flexibility | 32 (20.51%) | 7 (8.86%) | 25 (32.47%) | 0.001* |
| Withdrawal | 128 (82.05%) | 58 (73.42%) | 70 (90.91%) | 0.006* |
| **P*＜0.05 | | | | |
|
